# Creating Neuroscientific Knowledge Organization System Based on Word Representation and Agglomerative Clustering Algorithm

**DOI:** 10.3389/fninf.2020.00038

**Published:** 2020-08-18

**Authors:** Cunqing Huangfu, Yi Zeng, Yuwei Wang

**Affiliations:** ^1^Research Center for Brain-Inspired Intelligence, Institute of Automation, Chinese Academy of Sciences, Beijing, China; ^2^University of Chinese Academy of Sciences, Beijing, China; ^3^Center for Excellence in Brain Science and Intelligence Technology, Chinese Academy of Sciences, Shanghai, China; ^4^National Laboratory of Pattern Recognition, Institute of Automation, Chinese Academy of Sciences, Beijing, China

**Keywords:** literature analysis, clustering, word embedding, neuroscience, knowledge oganization system

## Abstract

The literature on neuroscience has grown rapidly in recent years with the emergence of new domains of research. In the context of this progress, creating a knowledge organization system (KOS) that can quickly incorporate terms of a given domain is an important aim in the area. In this article, we develop a systematic method based on word representation and the agglomerative clustering algorithm to semi-automatically build a hierarchical KOS. We collected 35,832 research keywords and 11,497 research methods from PubMed Central database, and organized them in a hierarchical structure according to semantic distance. We show that the proposed KOS can help find terms related to the given topics, analyze articles related to specific domains of research, and characterize the features of article clusters. The proposed method can significantly reduce the manual work required by experts to organize the KOS.

## Introduction

As the literature on neuroscience continues to grow, new subjects of research continue to emerge. Researchers need to identify these new topics and adapt to the changing landscape of the discipline. Knowledge organization system (KOS) “are used to organize materials for the purpose of retrieval and to manage a collection” (Hodge, 2000). KOS with hierarchical categorizations, like subject headings and taxonomies, can be useful to this end. Each node or branch in the hierarchical structure has an explainable meaning that can help researchers navigate through the taxonomy tree of the KOS level by level until they find their desired topic. A comprehensive KOS can also provide researchers with lists of genes, brain structures, research methods, behavior paradigms, cognitive functions, and diseases. It can thus serve as a valuable strategic space for researchers to choose methods and areas of research.

Medical Subject Heading (MeSH) is among the most prominent KOS in biomedical science. It is a massive collection of terms organized in a hierarchical manner that are used as recommended keywords for articles in the life sciences and medical research. It consists of a “new and thoroughly revised version of lists of subject headings compiled by NLM for its bibliographies and cataloging” (Lipscomb, 2000, p. 265). It has been used to search the medical literature (Coletti and Bleich, 2001), visualize research trends (Yang and Lee, 2018), extract knowledge (Brown and Patel, 2016), analyze the evolution of research terms (Antonio and Stefan, 2018), and cluster the scientific literature (Minguet et al., 2017). In the MeSH browser^1^, one can find terms representing all subdomains of research in the area. Other, similar, KOS include Disease Ontology (DO) (Schriml et al., 2012) and Gene Ontology (GO) (Ashburner et al., 2000). All these KOS were created manually or extracted from structured data.

It is important for researchers that the KOS be updated extensively and quickly. However, manual organization requires large amounts of time and labor, which indicates that a manually organized KOS cannot both update quickly and contain a comprehensive list of the relevant terms. According to Gläser et al. (2017), “detecting emerging topics would be a different matter again because a few, small special topics would need to be identified.” Manual organization also limits the number of terms that can be included in the system. In MeSH, the node “cognition”^2^ contains only 11 terms, far fewer than the actual number of keywords related to cognition.

Automatic and semi-automatic methods have been developed to organized terms based on the given corpus. Previous work has combined word representation with clustering algorithms. Word2vec (Mikolov et al., 2013) is among the most popular word representation training algorithms. It is a self-supervised deep learning model that can learn features of the co-occurrence of words and assign a specific “word vector” to each to represent its feature. If two words have similar semantics, such as “histological staining” and “histology analyses,” they always appear in similar contexts and, in the word2vec model, have similar word vectors. Thus word2vec representation can be used to depict the underlying semantics of terms. We can easily find terms with similar semantics by looking for terms with similar representations. We do not need to go over the entire term list or know the meaning of every single term. Doc2vec (Le and Mikolov, 2014) can simultaneously train document representations and word representations. The glove algorithm (Pennington et al., 2014) generates word representations in a similar way. Combining a word representation training algorithm with clustering algorithms like the k-means classifier can organize terms into clusters (Hu et al., 2018; Zhang et al., 2018; Duarte-Garcia et al., 2019; Onan, 2019; Zhou et al., 2020). Viegas et al. (2019) used clusters of words as “meta-words” to enhance document representation. These methods can reduce the amount of manual work required to organize the KOS, and many of them can yield a KOS that is explanatorily useful. However, the clustering algorithms used in the above methods cannot generate a KOS with a hierarchical categorization, as MeSH is.

Creating KOS automatically using word representation and clustering algorithm has another problem to be overcomed: the clustering result is not necessarily accordant with semantic groups. On one hand, the criteria of semantic groups are vague, which makes it challenging to develop objective criteria. Some words are core members of a given semantic group while others are marginal members. On the other hand, Hu et al. (2018) have also noted that in different semantic groups, the similarity thresholds of word representation are different and need to be manually determined. This is particularly germane to embedded semantic groups. In research using the k-means or other clustering algorithms, the criteria for semantic groups are controlled by the parameters of the algorithm – for example, the number of clusters or threshold of similarity. These criteria are applied to all clusters whereas different semantic groups have different thresholds of intrinsic similarity, which means that they cannot help adjust the accurate range of semantic groups.

A qualified clustering algorithm should have two features: First, it should be able to create a hierarchical structure according to the similarity of words. Second, it should allow us to manually adjust the similarity threshold for word representation while minimizing the manual work required. We think that the agglomerative clustering algorithm (Chidananda and Krishna, 1978) can fulfill this need. It organizes data nodes into a binary clustering tree according to their similarities. It finds the two globally closest data nodes, merges them into one, and again merges the two subsequently closest nodes until all nodes have been merged into a root node. Almost no parameter needs to be configured in this algorithm. The binary tree is a natural preliminary KOS with a hierarchical categorization. Around the root node, the distance between nodes is large, while around the tip of the tree, it is small. In this way, the similarity thresholds are automatically configured.

**FIGURE 1:**
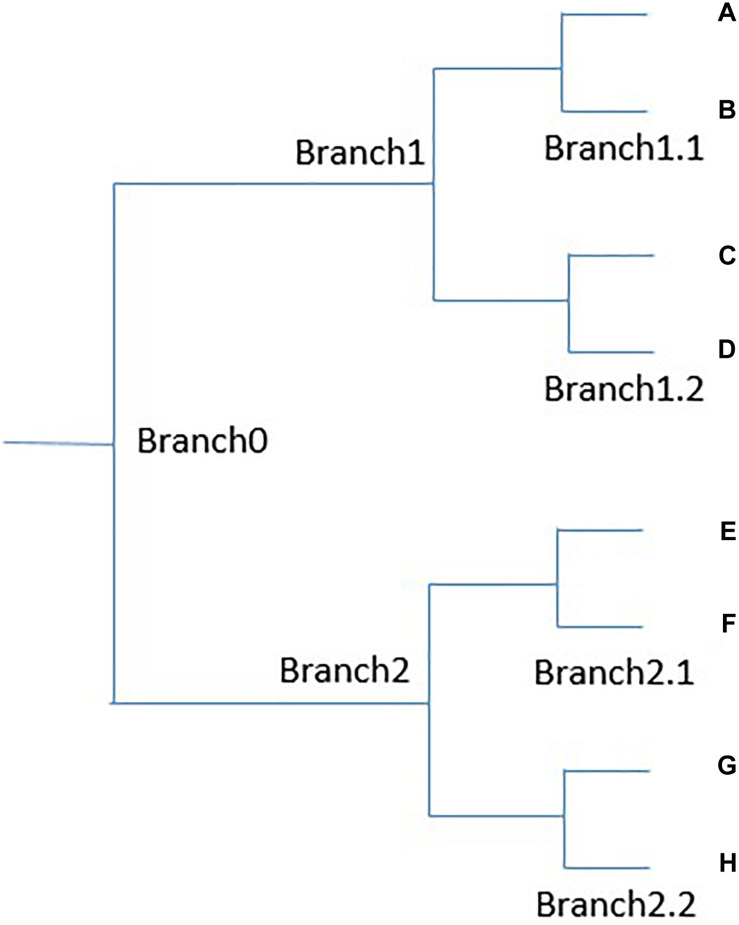
An example binary tree.

We devise a method to significantly reduce the manual work required to pick nodes with explainable meanings and, in this way, adjust the similarity threshold for word representation. For example, as shown in Figure 1, suppose a binary tree is generated by the agglomerative clustering algorithm. Leaf nodes A and B are most similar, C and D are less similar to A, and E–H are less similar still to A. If we know that A and B belong to the same semantic group, and want to know whether terms in branch 1 all belong to the same semantic group, we do not need to check both C and D, and usually need to check only one of them. Because the distance between A and C is usually larger than that between C and D, if A and C are in the same group, it is very likely that A and D are also in the same group. For the same reason, if we know that ABCD are in the same semantic group, and want to know whether EFGH are in the same group, we do not need to check all of EFGH, and instead need only check a few, such as E and G. Because the semantic distance between A, and E and G is greater than that between E and F, if A, and E and G are in the same semantic group, A and F are probably in it as well. For a small binary tree as in Figure 1, this mechanism may not seem very useful. However, in our clustering tree, one branch may contain hundreds or thousands of terms, and only a small fraction of them need to be manually checked to determine the meaning of a given node. The agglomerative clustering algorithm is the only available algorithm that can generate both a hierarchical structure and allow us to manually adjust the range of semantic groups.

Based on the idea describe above, we created a KOS for neuroscience containing 11,497 research methods and 35,832 keywords. We trained their vector representations based on PubMed Central (Gamble, 2017), containing full-text corpora, and clustered them using the agglomerative clustering algorithm. We chose 277 nodes from the clustering tree containing research methods and 378 from that containing keywords with explainable meanings. Most terms were covered under these nodes. This KOS provided us with a comprehensive landscape of research in neuroscience. Using this system, neuroscience researchers can quickly find the research domain of interest and obtained detailed lists of the relevant terms, like synonyms, genes, diseases, brain structures, and cognitive functions and tasks. Previous research has also provided similar epistemological landscapes of neuroscience. Yeung et al. (2017) summarized the most popular keywords in neuroscience on an annual basis. However, the objects of their study were single keywords, and they did not create a KOS that can help us understand research in neuroscience at different hierarchical levels. They also extracted only keywords that had appeared more than 100 times. Buchan et al. (2016) focus on 100 researchers in the area, and clustered and analyzed them. However, the clustering was based on social relations between the researchers, and territorial factors thus strongly influenced the results. Work by most of the chosen researchers was also dedicated to only a few popular domains of research, and lesser-known areas were not considered.

We then assessed the proposed KOS on two tasks related to the analysis of scientific literature. First, to show that the KOS could retrieve certain categories of articles, we investigated the relationship between research methods and keywords in neuroscience. The strategic space for researchers can be described simply as one where appropriate methods are applied to the corresponding research domains. Applying a new approach to a known subject of research, and applying an available method to a new research topic are two of the most convenient ways to design novel research. The adaptability between the clustered research methods and keywords is apparent at a glance.

Second, to show that the KOS can help describe the features of article clusters, we divided articles related to learning and memory into seven clusters and analyzed features of each using the KOS. Many methods are available to cluster documents, but it is challenging to depict features of specific clusters. Viegas et al. (2019) claimed that the clustered words (meta-words) can enhance the representation of documents. Term clusters contain terms with similar semantics, and their collective meaning can better reflect the semantic components of articles. In the KOS are clusters containing terms related to different topics, including the structures of organism, research methods, functions, and diseases. Each cluster represents a specific research topic. We analyzed article clusters by extracting all terms from them and determining the term cluster in the KOS to which they belonged. The related term clusters could then characterize features of the article clusters.

**FIGURE 2:**
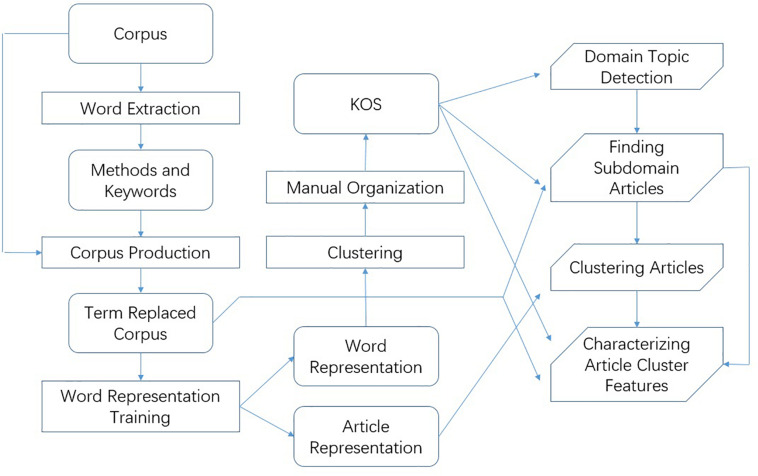
Flowchart of the proposed method.

In summary, we first develop a systematic procedure to semi-automatically organize a KOS with a hierarchical categorization of the neuroscience literature. The KOS can be used to retrieve articles related to certain research domains and characterize their features. Figure 2 shows a flowchart of the method.

## Materials and Methods

### Collecting Articles in Neuroscience

All articles were downloaded from PubMed Central (PMC) using the search keywords “brain,” “neural,” and “neuron” on October 8, 2018. The data were downloaded using the site’s advanced search function^3^, and all fields of all available articles, published from January 1, 1000, to October 8, 2018 were scanned for the keywords. On the search results’ page, we used the “Send to” function to send the results to file. The file format was set to XML and the sort order to “default.” A total of 613,184 articles were hence acquired.

### Word List Extraction

The keywords were extracted from the keyword section of the XML files extracted from PMC. The term list of research methods was extracted from the “Materials and Methods” sections of the scanned articles. The subtitles in this section usually featured names of experimental methods, and thus they were extracted as the names of research methods. The “Materials and Methods” section was identified by finding the word “method” or “procedure” in the title. Section titles like “Methods,” “Experimental procedures,” and “Methodology” were all thus identified as identical to the “Materials and Methods” section. Some words or phrases appear both in the keyword list and in the methods’ list. We would like to analyze the correlation of keywords and methods in articles, so we need to avoid duplicated terms. We deleted these terms from the keyword list.

All words and phrases were converted to lowercase. Each word or phrase in the list was assigned a number according to its ranking in terms of frequency. The most frequently occurring keyword was “hippocampus,” and was assigned the ID kwd1. The most frequently occurring method name was “statistical analysis,” and was assigned the ID mtd1.

### Word Embedding Training

All the articles collected were transferred to a corpus for word embedding training. Words and phrases are recognized from natural language in a “longer phrase-first” manner. For example, from the sentence “visual short-term memory for high-resolution associations is impaired in patients with medial temporal lobe damage.” we recognized the embedded keyword phrases “visual short-term memory” (kwd5577) and “short-term memory” (kwd1284). However, the keyword “short-term memory” was not counted because the longer phrase “visual short-term memory” had a higher priority. This setting was chosen because embedded phrases would have otherwise interfered with word embedding training. If the embedded phrases were all listed out in the training corpus, “image analyses” and “gene analyses” would have similar word embeddings, for instance, because they are both related to “analyses.” The training algorithm would capture shallow semantic relations rather than deeper ones. All other words not recognized as keywords or methods were discarded. Not many of these remained at the end of the training, except for stop words.

We used the Doc2Vec (Le and Mikolov, 2014) function in the sklearn (Pedregosa et al., 2012) package in Python 3.6.5. The Doc2Vec algorithm is known to be the most effective at generating article representations (Curiskis et al., 2019). The window size was set to seven and the vector size was 300. The minimum word count was 10. The number of training iterations was 10. These parameters were taken from Shahmirzadi et al. (2018). The learning rate was set to 0.025 and reduced by 0.002 in every epoch. The model parameter “dbow_words” was set to zero by default so that the Doc2Vec model could simultaneously train word vectors in skip-gram mode. Doc2Vec was used instead of Word2vec because it can generate word representation as well as document vectors. The Doc2Vec model package used was gensim (Řehůřek and Sojka, 2010).

### Word Clustering

The keywords and methods were clustered separately using the agglomerative clustering algorithm (Chidananda and Krishna, 1978).

When calculating the distances between clusters, the agglomerative clustering algorithm has three kinds of linkage calculating algorithms: single linkage, complete linkage, and average linkage. Yim and Ramdeen (2015) have shown that the that single-linkage algorithm suffers from outliers, and may group together clusters that are far apart through chaining nodes, whereas “complete linkage does not necessarily merge groups close together owing to outlying cases that may be far apart,” and “average linkage represents a natural compromise between single linkage and complete linkage.” Thus we chose the average linkage algorithm for agglomerative clustering.

The word vectors were normalized so that the norm was one. Keywords appearing more than five times in the keyword section of the articles, and more than 10 times in the corpus were used for further analysis. Research methods that had appeared more than two times in the “Methods” sections of articles, and more than 10 times in the corpus were also used. If a term appeared rarely in the corpus, it meant that the word representation might not have been trained well and introduced noise to the subsequent steps of clustering. The keyword tree and the method clustering tree were fully printed in Supplementary Materials S1, S2.

Each branching node in the tree was labeled by a number based on the node-labeling function in the sklearn package. If there were n terms in the tree, n-1 branching nodes were needed to merge them with the root node. Node numbers 1 to n were used to label the leaf nodes representing the terms. When the first two nodes were merged together to form a new branching node, this node was labeled as n+1. When two more nodes merged to create yet another branching node, it was labeled as n+2. There were a total of 38,036 terms in the keyword clustering tree and 12,488 terms in the method clustering tree. The root node in the keyword clustering tree was labeled as 76,072, 2 × 38,036.

The clustering tree was manually organized into 378 keyword clusters (Supplementary Material S3) and 277 method clusters (Supplementary Material S4) under the following guiding principles: A cluster must have a specific explainable meaning so that it may represent a subdomain of research in neuroscience. The granularity of the cluster was adjusted such that (1) for methods and keywords, 300 clusters were acquired in total, and (2) all clusters were monophyletic branches in the binary clustering tree. The second setting was used because a monophyletic branch structure is easy to maintain. The 378 keyword clusters contained 35,832 keywords, and the 277 method clusters contained 11,497 methods. Some terms were not included in the hierarchical structure, including abbreviations with explanations, terms that were too general, or terms that were poorly organized. For example, some nodes contained such names of methods as “method 1” and “procedure 2.” They were also used as subtitles in the “Materials and Methods” sections, but are not known names of research methods. They are grouped together and discarded from the KOS.

Sometimes, when the paraphyletic branch of a clustering tree was more likely to belong to one cluster with a consistent explainable meaning, we gave the same cluster name to both the small monophyletic branches in the paraphyletic branch.

The results of the clustering of methods and keywords were reorganized into a more general, level-3, cluster result (Supplementary Materials S5, S6).

### Visualizing Clusters of Keywords and Research Methods

For each cluster of keywords and research methods, the average normalized word vector was calculated as its representation. The results of visualization were calculated by the TSNE (Hinton and van der Maaten, 2008) algorithm in the sklearn package (Pedregosa et al., 2012) based on these representations (Figures 4, 5). The area occupied by the dots was proportional to the total citations of the articles in PubMed for a given term cluster. The colors of the dots represent the level-3 superclusters of the given cluster, which is marked in the legend. Clusters with similar meanings had similar colors.

### Combination of Heat Maps of Neuroscience Articles Over Clusters of Methods and Keywords

We calculated the relation between the clusters of research keywords and methods by calculating their co-occurrence in the articles. If keyword “kwd1” in keyword cluster A for a times and method “mtd1” in method cluster B appeared b times in one article, the relation between clusters A and B for this article was a × b. We also considered the weight of an article (W) as its number of citations in PubMed. Suppose there are m keyword clusters in an article, and each has K_1_, K_2_, K_3_, …, K_m_ terms. Suppose also that there are n method clusters in the article, and each has M_1_, M_2_, M_3_, …, M_n_ methods. Then, for keyword cluster i and method cluster j, the correlation index on the given article is:

(1) $$C_{i\,j}=W\,\frac{K_{i}\,M_{j}}{\Sigma_{1<i<m,1<j<n}\,K_{j}\,M_{j}}$$

By adding the correlation indices of all articles together, we acquired the correlation index between all keyword clusters and method clusters. The log value of the matrix of correlation indices was visualized using a heat map (Figure 6). The color in the chart represents how often the corresponding method and keyword were combined in an article; the most correlated combination is in yellow while the least correlated combination is in blue.

### Analyzing Articles in PMC Related to Learning and Memory

All 12,391 articles containing at least one keyword from the cluster “learning and memory” were selected. Their representation as calculated by Doc2Vec was obtained from the genism word embedding model. The articles were clustered using the affinity propagation algorithm (Frey and Dueck, 2007) in the sklearn package with the default settings. We used the affinity propagation clustering method because it does not require a large number of parameters, such as the number of clusters.

## Results

### Neuroscience Knowledge Organization System

#### Binary Clustering Tree

**FIGURE 3:**
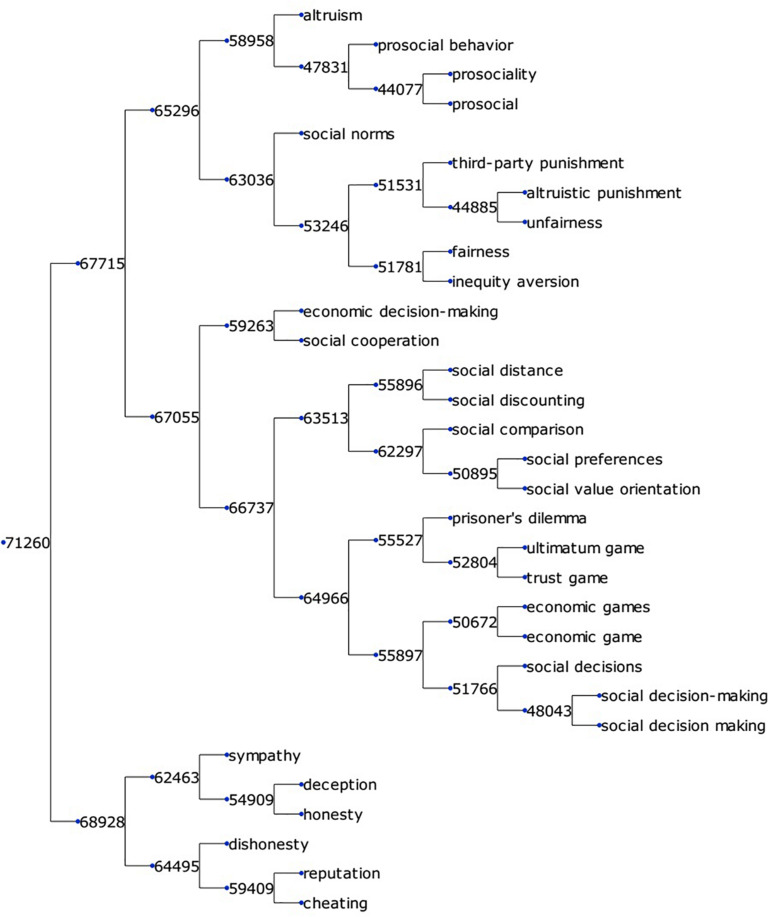
A small fraction of the keyword clustering tree. The binary tree is visualized using the Python package ETE3 (Huerta-Cepas et al., 2016).

To create a comprehensive KOS with hierarchical categorization for neuroscience, we extracted 35,832 keywords, and 11,497 research methods, calculated word vector representations for each, and generated a binary clustering tree. Each node in given a digit as its ID, like 71,260. The meaning of the ID is explained in section “Word Clustering.” Figure 3 shows a small fraction of the keyword clustering tree extracted from branch node 71,260 in Supplementary Material S1. This fraction of terms was related to social psychology. As mentioned above, the binary tree can help us determine whether two of its children belong to the same semantic group. Figure 3 shows that the binary tree was accurate enough to perform this task. Node 65,296 contained four terms – “altruism,” “prosocial behavior,” “prosociality,” and “prosocial” – belonging to the semantic group “prosociality.” Its sibling node 63,036 contained terms like “fairness,” “unfairness,” and “social norm,” which did not belong to the semantic group “prosociality.” Node 67,055 contained terms like “social distance” or “social cooperation,” and can be summarized as a semantic group of “social experiment paradigms.” Node 67,055, as well as its sibling 65,296 belonged to the same semantic group of “social research.” In the binary tree, terms were organized according to their semantic distances. Similar terms were clustered into the same branch and, by selectively checking the term in a branch, we could easily determine the contents of terms in the entire branch. Also, by going up and down the hierarchy, we could zoom in and out over a range of semantic meanings to choose the granularity of the semantic groups.

#### Method and Keyword Clusters

The hierarchical binary tree can organize terms according to distance, but cannot directly serve as a KOS because not all nodes in it have explainable meanings. For example, if a tree node contains 20 chemical receptors with similar functions, there would be 19 intermediate tree nodes in the binary tree, but 19 explainable meanings to categorize these receptors are unlikely to be available. Some terms were simply parallel to one another. To better reveal the structure of the clustering tree, we chose 378 nodes of the keyword tree and 277 of the method tree. Each cluster contained a group of keywords or methods with specific, explainable meanings, and most of them represented a subdomain of research. Each cluster was a monophyletic tree in the clustering, and was given a name to describe its contents. Some clusters, like the method cluster 24,328, “methods,” in line 166 of Supplementary Material S4, contained general titles in the “Methods” section of an article, like “protocols” and “methodology,” and did not represent a specific research domain. The 378 keyword clusters and 277 method clusters were further clustered into 29 and 25 level-3 clusters, respectively, using the same method. This clustering system is beneficial for neuroscience researchers for finding terms related to a topic in which they are interested. The entire binary clustering tree of keywords and methods is presented in Supplementary Materials S1, S2. The 378 keyword clusters and 277 method clusters are shown in Supplementary Materials S3, S4, respectively. The 29 level-3 keyword clusters and 25 level-3 method clusters are presented in Supplementary Materials S5, S6, respectively. The same label for nodes of the keyword tree in Supplementary Materials S1, S3, S5 represents the same node, and the same label of nodes in the method tree in Supplementary Materials S2, S4, S6 represents the corresponding node.

#### Landscape of Neuroscience

**FIGURE 4:**
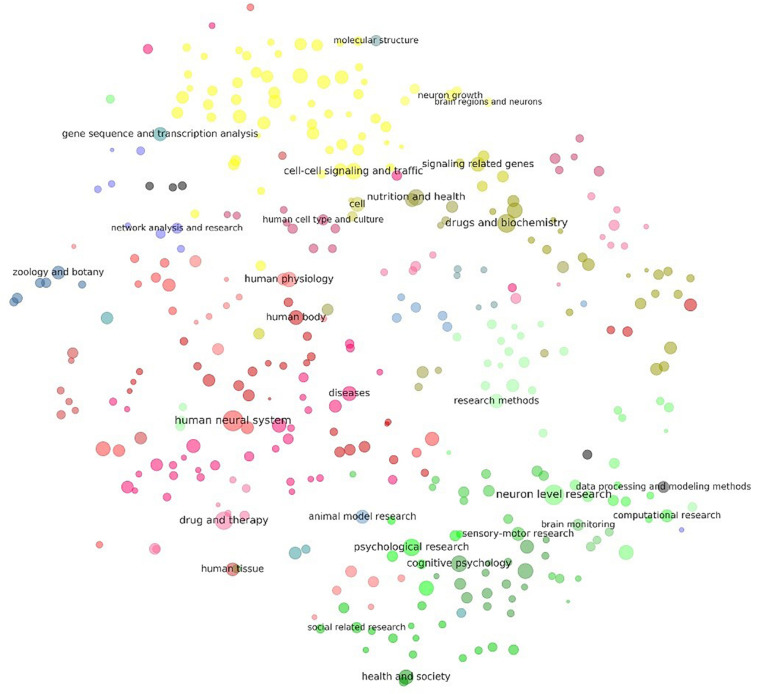
Visualization of the results of clustering of keywords related to neuroscience research. Each dot in the above figure represents a keyword cluster. The size of the dot represents the total number of references to the corresponding keywords in the cluster. The color of the dot represents level 3 of the cluster. The name of the cluster is provided on the largest dot under the level-3 cluster. Dot coordination was determined by the average word vector of terms in the cluster as calculated by the TSNE algorithm. More detailed results of the visualization of the same layout are given in Supplementary Material S7, where all cluster names are tagged.

**FIGURE 5:**
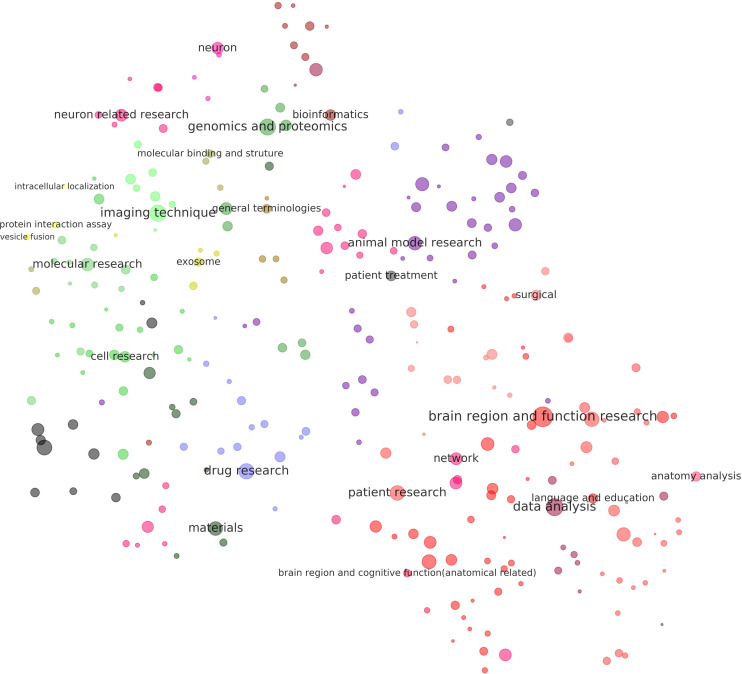
Visualization of the results of clustering of methods related to neuroscience research. Each dot in the above figure represents a method cluster. The size of the dot represents the total number of references to methods in the cluster. The color of the dot represents level 3 of the cluster. The name of the cluster is provided on the largest dot under the level-3 cluster. Dot coordination was determined by the average word vector of terms in the cluster as calculated by the TSNE algorithm. More detailed results of the visualization of the same layout are given in Supplementary Material S8, where all cluster names are tagged.

Figures 4, 5 visualize the clusters of keywords and methods, respectively. Dots with similar colors tended to aggregate together, which means that the results of the clustering algorithm and the visualization algorithm were coherent. These two figures can be considered a simplified atlas of neuroscience, in which subdomains have been elaborated, and the relations among the subdomain, number of references, and relative relations are shown.

Figure 4 shows that there were two poles in the graph: One was cognitive psychology (green), surrounded by neuron-level research, research on animal models, and related social research. The other pole was cell–cell signaling and traffic (yellow), surrounded by gene sequence and transcription analyses, molecular structure, and genes associated with signaling. Medical research (mostly in red and purple) was in the middle and left, combined with keywords specifically related to humans. Keywords related to research methods and bioinformatics were in the middle-right of the graph.

Figure 5 shows that methods, like keywords, had two poles: One investigated cognitive functions while the other studied molecules and cells. The center of the first pole identified regions of the brain and functional research, surrounded by anatomical analysis, language and education, and networks. The core of the second pole was neuron-related research, surrounded by genomics and proteomics, the binding and structure of molecules, and molecular research. Methods related to animal models and electrophysiology were in the top right.

#### Comparison With MeSH

To measure the accuracy and completeness of our categories of terms, we compared our clustering tree with the MeSH ontology tree. In most domains related to neuroscience, our collection of terms was more detailed than that of MeSH. For example, when searching “glutamate” in MeSH, only one result, “Glutamic Acid” [D12.125.427.300], representing the chemical itself, is obtained. But in our collection of keywords, 60 terms were found referring to glutamate, which belonged to several clusters, including cluster 75,631 (“addiction, psychoactive drugs, and reward system”), cluster 75,552 (“synapse”), and cluster 74,742 (“glutamate”). The MeSH category “Learning” [F02.463.425] contained 115 terms (synonyms were also counted), whereas our keyword branch 75,751 (“learning and memory”) of the clustering tree contained 565 terms. Neuroscience research is highly dependent on lists of certain kinds of proteins, genes, and brain structures. Thus, a detailed and well-structured list can be valuable for researchers.

We collected all terms related to regions of the brain in MeSH 2019 to determine whether their presence and location in the ontology tree. Terms associated with regions of the brain were collected under the category of “Brain” [A08.186.211]. Of the 77 terms collected from MeSH, 16 (21%) were not found in our term list, 37 (48%) belonged to the category “brain regions, circuits, and neurons,” and the other terms were scattered in other categories.

The key difference between our KOS and MeSH is that nodes of the latter can have multiple categories. For example, the MeSH node “Glymphatic System” [A08.186.211.150] is both a part of the brain and the lymphatic system. In our KOS, it was classified into the category “interstitial fluid and lymphatic system,” whereas it was under the nodes of the Brain [A08.186.211], Lymphatic System [A15.382.520], and Cardiovascular System [A07] in MeSH. Similar examples include “Olivary Nucleus,” which was classified into “hearing related brain structures” in our system but under the node Medulla Oblongata [A08.186.211.132.810.591.500] in MeSH. This brain structure is closely related to the hearing function, and is not categorized into “brain regions, circuits, and neurons” in our system. The structure of the binary tree in our KOS thus could not incorporate all relations between tree nodes.

We conclude that our method can incorporate more terms and is more convenient to update than MeSH. However, although the binary tree can help organize terms according to similarity, the hierarchical structure cannot incorporate all relations in the semantic space. Manual work or an advanced algorithm is required to excavate all relations between nodes of the binary tree. The method used here alone cannot generate a KOS to replace MeSH, but can provide a surplus of new and well-organized terms for people building a KOS like MeSH. It can also help build a preliminary KOS quickly without requiring large amounts of manual work.

### The KOS Can Be Used to Identify Articles Related to Certain Research Domains

Scientists, especially bibliometric scientists, need to locate articles in certain research areas. The term clusters in the KOS can be used as search keywords to find articles. In this and the following section, we perform scientific literature tasks using the KOS to demonstrate that although the KOS is semi-automatically created with much less efforts, it can perform function just like other KOS. Here we demonstrate that the KOS can retrieve articles related to certain research domains.

#### Investigating the Relationship Between Research Methods and Keywords

**FIGURE 6:**
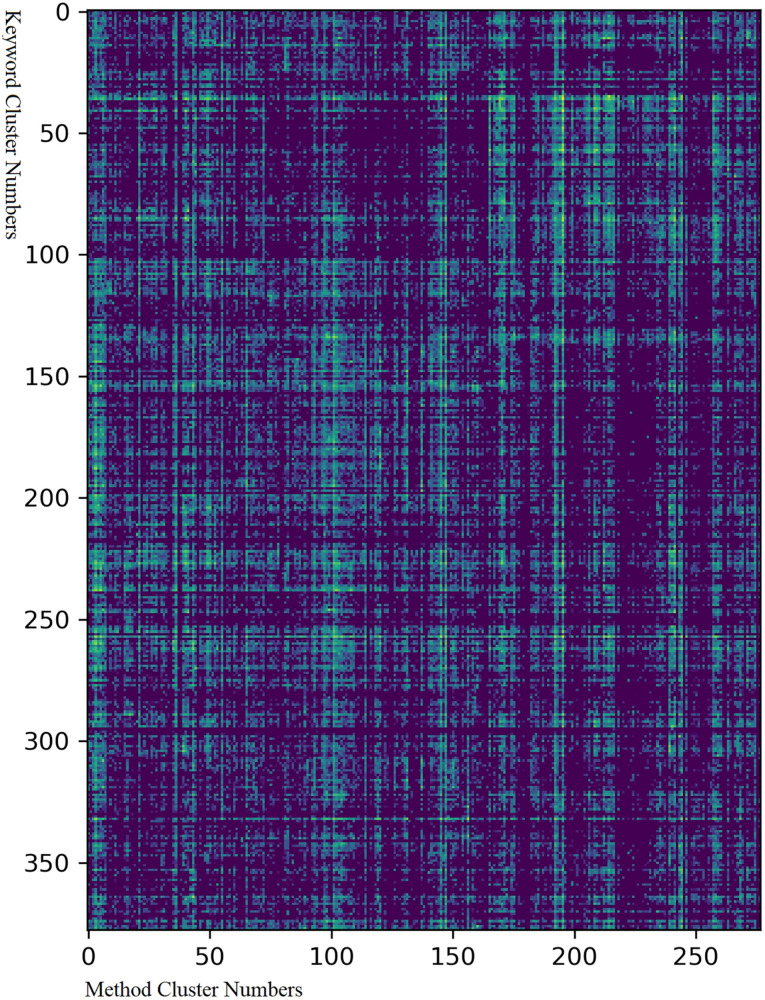
Matrix of combinations between clusters of keywords and methods. The Y-axis represents the number of keyword clusters from 1 to 378 while the X-axis the number of method clusters from 1 to 277. The corresponding data are provided in Supplementary Material S7. The colors represent how often a method and a keyword cluster appeared together and were cited in one article: yellow means that this happened very often while deep blue means that it never happened.

The strategic space for researchers is centered around the research methods they can use and the topics in which they are interested. A comprehensive analysis of the adaptability between research methods and keywords can help researchers expand their strategic space for research. To analyze the relationship between the research methods and the keywords, we calculated the co-occurrence index of every research method and keyword. The resulting relation matrix is visualized in Figure 6, and the data are provided in Supplementary Material S9.

Figure 6 shows the following: (1) There were dense and sparse areas in the graph, which means that certain groups of methods were applicable or not to specific groups of keywords. (2) Some keyword/method clusters were very widely adaptable to almost all method/keyword clusters, like keyword cluster 259 (“neurodegenerative disease”) and method cluster 147 (“animal model”). (3) Fine structures were observed in the graph, which means that the relationship between methods and keywords was complex. (4) In general, the research methods and the keywords were extensively correlated except in a few sparse areas. The sparse areas in keyword clusters 40–70 and method clusters 100–150 were combinations of the keywords of cognitive function and cell-related research methods, and those in keyword clusters 140–220 and method clusters 200–250 were combinations of keywords related to cells and research methods relevant to EEG\brain imaging. Uncommon popular combinations appears in this area, such as the combination of the keyword “neurotrophin and receptors” and the method “auditory analyses.” This is because that although neurotrophin is primarily related to cell-level research, it is also used to treat hearing loss, while auditory analysis is a method to assess auditory capabilities. Although the cell-level operation is not commonly related to cognitive function, with developments in the transfection virus and cytotherapy, these obscure domains may become relevant.

The three most commonly referenced method clusters were “behavioral and cognitive tasks,” “cell survival,” and “animal model.” The three most commonly referenced keyword clusters were “brain regions, circuits, and neurons,” “neurodegenerative disease,” and “biochemistry.” The 20 most commonly referenced combinations of the clusters of keywords and methods are listed in Table 1.

**TABLE 1:** Twenty most common combinations of the clusters of methods and keywords.

**Correlation index**	**Method**	**Keyword**
2.81E+04	Behavioral and cognitive tasks	Brain regions, circuits, and neurons
2.03E+04	Behavioral and cognitive tasks	Learning and memory
1.53E+04	Behavioral and cognitive tasks	Cognitive abilities
1.17E+04	Behavioral and cognitive tasks	Neurodegenerative disease
9.93E+03	Image analysis	Brain region analysis
8.03E+03	Irradiation and DNA damage	Cancer
7.77E+03	Behavioral and cognitive tasks	Mental disease, mental stress, and related terms
7.25E+03	Conditioning-related mental disease	Addiction, psychoactive drugs, and reward system
7.19E+03	Brain and muscle injury	Injury, organ damage, and treatment
6.57E+03	Imaging methods	MRI and PET imaging techniques
6.46E+03	Cell survival	Cognitive and emotional impairment
6.17E+03	Behavioral and cognitive tasks	Addiction, psychoactive drugs, and reward system
5.90E+03	Behavioral and cognitive tasks	Emotion and bias
5.90E+03	Behavioral and cognitive tasks	Sex response
5.79E+03	Immune staining and histology	Neurodegenerative disease
5.79E+03	Mental status	Brain regions, circuits, and neurons
5.77E+03	Behavioral and cognitive tasks	Visual cognition
5.59E+03	Mental status	Genes closely related to sequence and transcription analysis
5.56E+03	Imaging methods	Neurodegenerative disease
5.15E+03	Expression analysis	Sequence and transcription analysis

The research method “behavioral and cognitive tasks” occupied a significant position in this table. Of the 20 combinations, nine involved “behavioral and cognitive tasks.” It also ranked first in terms of the number of citations. It contained 200 terms, compared with an average number of 41 terms in each cluster, as well as experiment paradigms like “change detection task,” “line bisection task,” “Iowa gambling task,” and related terms like “sensitivity to reward,” “learning sessions,” and “experimental paradigm.” These methods examine the cognitive performance of organisms, mostly human beings. The term “experimental paradigm” seems not explicitly related to behavioral and cognitive tasks, but refers to experimental methods as “experimental paradigm” is indeed the preferred expression in this subdomain.

#### Correlation Index Follows the Power Law

**FIGURE 7:**
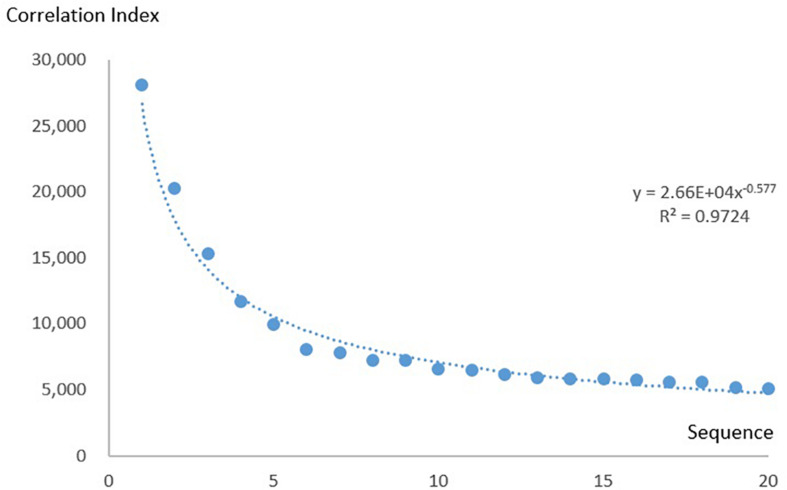
Correlation index of the 20 most commonly used combinations.

**FIGURE 8:**
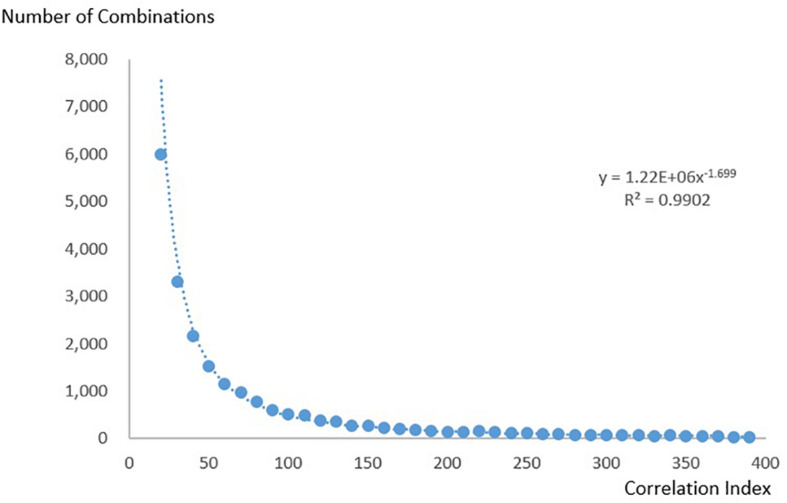
The relation between the correlation index and the number of combinations.

To show that the KOS can be applied to bibliometric research, we present the following case: We plotted the correlation index of the 20 most commonly used combinations against their sequences and found that they followed a power law distribution (Figure 7). We then investigated whether the law also applied to combinations with lower correlation indices. We calculated the number of combinations using correlation indices from 10 to 400 at intervals of 10. Figure 8 shows the relation between the correlation index and the number of combinations. The result follows a power law distribution as well.

Combinations with correlation indices lower than 10 were discarded because they were large in number. Combinations with correlation indices higher than 400 were also discarded because the long tail caused the number to become unstable.

**FIGURE 9:**
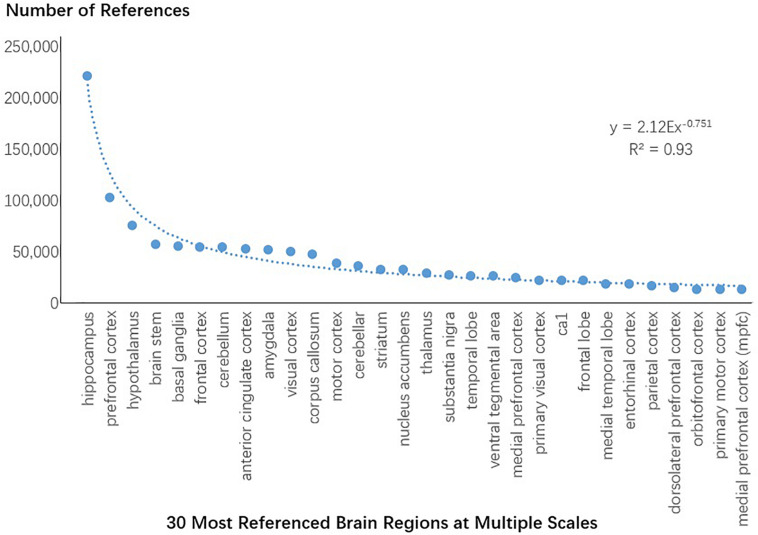
All citations of the 30 most referenced regions of the brain at multiple scales.

The research domain investigated above was defined by our categories of research methods and keywords. However, the scope of these categories was manually adjusted, because of which the size of the categories might have contained subjective factors. We thus collected terms related to regions of the brain and calculate whether all references containing these terms also followed the power law. The terms related to regions of the brain were extracted from the branches 73,705 and 75,966 of the clustering tree for keywords and branch 16,444 of the clustering tree for methods. A total of 780 terms were obtained. The articles containing these terms were retrieved and all total reference to these articles were obtained from PubMed. Using these data, we calculated the total number of references to each term related to regions of the brain and plotted the 30 most referenced regions of the brain (Figure 9). Synonym reference counts were merged. The distribution of references to regions of the brain also followed the power law, and the most referenced region was the hippocampus.

The power law distribution of the correlation index suggests that the Matthew effect has been significant in the development of neuroscience (Merton, 1968; Price, 1976). Successful research domains defined by a combination of research methods and research keywords become popular, draw more attention, and the combination itself finally becomes more popular.

Most combinations between keywords and methods have received little attention, perhaps because popular research topics are more likely to attract new research. Using the KOS created here, researchers can find appropriate methods for subjects of their interest as well as promising issues to which their own research methods can be applied.

### KOS Can Characterize Features of Article Clusters

A feature of an article, such as the method it uses, the topic it discusses, or the problem it intends to solve, can be depicted by its keywords and methods. Viegas et al. (2019) used “meta-words” (clustered words with similar semantics) to depict the features of articles. The term clusters in our KOS have specific explainable meanings, and most represent a subdomain of neuroscience, where this can be used to describe the features of articles. Here we demonstrate that the KOS can help characterize features of articles.

Suppose we are interested in research in “learning and memory.” We want to know how many types of articles are there in the field and what their features are. We collected PMC articles containing terms in the keyword cluster “learning and memory,” acquired the document vector in the Doc2Vec model, and clustered them using the affinity propagation clustering algorithm (Frey and Dueck, 2007). The first round of clustering produced 686 clusters that were again clustered in the second round using the same algorithm to generate 51 clusters. The third round produced seven clusters. For each of these, the 10 most relevant clusters of keywords and methods were counted. The 10 most commonly cited articles in PubMed were also retrieved. The results are organized in Supplementary Material S8.

To depict features of the article cluster, we calculate the coverage of article groups on the term clusters. If an article contained at least one term in a term cluster, it was said to cover the term cluster. For a group of articles, we defined coverage over a term cluster as the percentage of articles that covered it. For example, if 60% of articles in the group contained at least one term in term cluster A, the coverage of these articles over A was 60%. If the coverage of a group of articles over a certain term cluster was high, this meant that these articles frequently mentioned terms belonging to the term cluster. By calculating and ranking the coverage values, we found the term clusters with the highest coverage values, thus revealing the features of the articles.

The sorted coverage vector of all articles related to learning and memory is listed in Table 2. In the clustering process, no expert knowledge was used and no parameter adjustment of the clustering algorithm was required. The clustering method was effective on a variety of article clusters. We applied this method to several clusters and produced similar results. Articles related to the term “olfactory” produced 15 clusters in the second round and three in the third round; articles related to the term “wound healing” produced 11 clusters in the second round and three in the third; those related to “acetylcholine” produced 26 clusters in the second round and five in the third round. This semi-automatic analysis can be applied to a variety of usage scenarios.

**TABLE 2:** Coverage of keywords and methods in all articles related to learning and memory.

**ID**	**Cluster name**	**Coverage**
**Most relevant keywords**		
131	Terms	83.42%
4	Data processing and modeling methods	81.81%
135	Cognitive elements description	81.38%
36	Brain regions, circuits, and neurons	72.18%
136	Cognitive elements	71.33%
3	Behavior	67.13%
102	Health-related social research	65.03%
5	Brain image quantitative methods	62.57%
326	Terms	61.37%
79	Machine learning	58.55%
**Most relevant methods**		
147	Animal model	83.42%
195	Behavioral and cognitive tasks	82.55%
193	Data	69.61%
192	Statistics	68.03%
72	Methods	61.42%
97	Animals, subjects, cell lines, ethics, and reagents	60.92%
198	Behavioral tests	56.84%
234	Statistical evaluations	56.64%
94	Methods	54.70%
21	Autonomous behaviors	54.06%

The coverage value is the percentage of learning- and memory-related articles containing terms related to the corresponding cluster. The keyword cluster “learning and memory” itself has been removed from the above list.

Table 2 shows features of articles related to learning and memory. In the keyword section, “brain regions, circuits, and neurons” were often involved in research, as were “cognitive behavior” and “machine learning.” We can conclude that many articles used animal models, and involved behavioral and cognitive tasks. More details can be acquired as well. For example, if we want to determine the regions of the brain, circuits, and neurons most related to learning and memory, we can calculate the coverage of a single term or a small group of terms in the keyword cluster “brain regions, circuits, and neurons.”

This investigation procedure can be applied to all seven sub-clusters. To highlight features of the articles in each sub-cluster, we subtracted the coverage vectors of all articles from vectors of articles in a given sub-cluster to generate a “coverage difference vector.” A large value of the coverage difference vector suggested that the corresponding term cluster was closely related to the given sub-cluster, but not very closely related to all articles related to learning and memory. This difference reflects what is highlighted in the sub-cluster. To show how we analyzed features of each article cluster, we arbitrarily use cluster 1 as an example. The 10 clusters each of keywords and methods with the highest coverage difference are listed to depict their features (Table 3 and Supplementary Material S10).

**TABLE 3:** Difference in coverage between clusters of keywords and methods on articles in cluster 1.

**ID**	**Cluster name**	**Coverage difference**
**Most related keywords**		
114	Insectology	63.59%
115	Invertebrate zoology	61.58%
112	Olfactory	52.08%
113	Taste	45.66%
104	Model organism	45.01%
116	Organism categories and evolution	43.36%
228	Nutrition and healthy food	38.17%
106	Florescent tools	36.22%
105	Mutant	36.09%
6	Immunohistochemistry techniques	34.87%
**Most related methods**		
9	Invertebrate model animals and behavior assays	58.57%
8	Odor experiments	38.69%
101	Expression analysis	33.21%
36	Animal model induction	29.61%
21	Autonomous behaviors	28.37%
49	Electrophysiology measurements	28.04%
184	Gene interaction and function analysis	24.31%
55	Live-imaging	22.28%
131	Cell proliferation and aggregation assays	21.14%
259	Training therapy	20.83%

Table 2 shows that most experiments in cluster 1 had been conducted on insects and other invertebrates, evident from the keyword clusters “insectology” and “invertebrate zoology,” and the method cluster “invertebrate model animals and behavior assays.” Moreover, olfactory and taste-related topics were popular in this cluster. The clusters “mutant” and “expression analysis” also indicate that gene-related research was popular in the relevant articles. Florescence tools had been prevalent in this kind of research.

To investigate whether this analysis is correct, we chose the three most commonly referenced articles in this cluster and manually analyzed their contents. The article titled “The neuronal architecture of the mushroom body provides a logic for associative learning” (Aso et al., 2014a) in this cluster had been referenced 182 times. It examined sense-related (odor) learning and the structure of the memory neuron in the brain of *Drosophila*, and used transgene methods and fluorescent tools. The article “Dietary choice behavior in *Caenorhabditis elegans*” (Shtonda, 2006) had been referenced 132 times. It investigated the feeding behaviors behavior of *C. elegans* to identify the underlying gene and neuron. The article “Mushroom body output neurons encode valence and guide memory-based action selection in *Drosophila*” (Aso et al., 2014b) had been referenced 121 times. It investigates the odor sensory function of MBON (mushroom body output neuron). The method it used includes odor stimulation experiments, transgene methods and fluorescent tools. We see that the names of terms in the cluster adequately characterized the feature of the relevant articles.

Using the same method, we investigated the other article clusters:

Cluster 2: Research on vertebrates at the molecular and cell levels:

This research (Supplementary Material S10, sheet 2) investigate neural system function of verterbrates from the perspective of molecular and cell level. Different from cluster 1, both low- and high-level cognitive functions were investigated because these functions are observed in vertebrates. The gene, molecule, neuron, and behavior are essential data in this kind of research. Most such research involves molecular- and cell-level experiments, and some studies have generated or utilized animal models of diseases.

Cluster 3: Behavioral and neuron-recording experiments:

This research (Supplementary Material S10, sheet 3) used behavior paradigms to investigate cognitive processes while recording neuronal activity and behavioral output. Most such experiments were performed on animals, especially monkeys and rats. The recording to behavioral and neuronal activities was the most critical data in the experiments. Changes in neuronal activity reflect plasticity, and thus plasticity-related conclusions were common in this kind of research. Many experiments involved surgery and recorded real-time brain signals. Through this kind of research, neurons responsive to specific types of cognitive functions were identified. The plasticity of neurons is usually investigated using learning- and memory-related task. Thus, the neurons learning particular kinds of information can be identified in this research.

Cluster 4: Circuit-related research:

This research investigated the relationship between neural circuits and cognitive functions, such as reward or fear conditioning. Some studies had been performed using human subjects but most had used other mammals. The goal of such research is to investigate the neural circuit underlying cognitive functions, and whether regions of the brain/neurons are related to cognitive processes formed the most critical data in experiments. Much of the significance was derived from a comparison between neural circuits related to different cognitive functions. Neurotransmitters or their receptors are sometimes used to mark neurons and regions of the brain, and receptor agonists are often used in experiments. We see that (Supplementary Material S10, sheet 4) the feature of this cluster is very similar to the features of all articles related to learning and memory, which means articles in this cluster can best represent the features of all articles.

Cluster 5: Cognitive neuroscience research:

This research investigated details of cognitive process using behavioral output and brain imaging. Subjects in the relevant experiment were required to perform a series of tasks as their behaviors and patterns of brain activity were recorded. Changes in the behavioral output and activities/areas of the brain were the most critical data in the experiment. The significance of this research was in decomposing cognitive process into smaller elements and identify regions of the brain to which they are related. We see that (Supplementary Material S10, sheet 5) the methods used in this cluster were mostly non-invasive methods that can be applied to human. “Participants” ranked first in the methods’ list, which means that most of this research had been carried out on humans.

Cluster 6: Psychological research:

This research investigated details of the cognitive process using psychological methods. We see that (Supplementary Material S10, sheet 6) keywords related to emotions and rewards were ranked high in the keyword list while psychological methods had a high rank in the methods’ list. Compared with cluster 2, this research is more closely related to diseases and behavioral performance is more critical. Interval-related research methods are prevalent in this cluster, perhaps because they are less invasive than those in other clusters.

Cluster 7: Sequence-related learning and memory:

This research (Supplementary Material S10, sheet 7) investigated the mechanism and influence of sequence learning, including motor, language, and sound sequences. Performance on sequence learning and memory-related tasks formed the most critical data in this research. Some studies had investigated mechanisms of sequence learning and others had examined the influence of other factors, such as sleep, on it. Some experiments had been conducted on humans and others on other mammals.

Thus, features of the article clusters can be adequately characterized by the KOS.

## Conclusion

### Summary

We first developed a systematic procedure to semi-automatically organize the KOS in a hierarchical categorization based on a corpus. We have shown that it can be used to retrieve articles related to certain topics and characterize features of article groups.

### Discussion

#### Word Representation, Clustering Method, and KOS

A hierarchical knowledge organization system (KOS) is difficult to build because it requires a large amount of manual work by domain experts. Our proposed method provides a fast and convenient way to build a KOS based on a corpus, which means that researchers can build their own KOS using their own versions of corpora. We showed that the KOS can perform many functions like other manually organized KOS: It can provide a full list of research domains with terms related to them, retrieve articles related to certain domains, and can be used to depict features of article clusters. A researcher need only follow the following steps to analyze a large corpus:

1. Extract terms from the corpus.
2. Train a word vector on the corpus.
3. Cluster terms according to word vector distance.
4. Investigate the clustering tree, and select branches containing terms related to a certain desired domain.
5. Use the terms to retrieve articles related to a given domain.
6. Cluster the articles.
7. Use the term clusters to depict features of article clusters.

This procedure does not require researchers to have extensive knowledge of algorithms, and does not demand model calibration. Our KOS is not as well organized as other KOS, like MeSH, but can reduce the amount of manual work needed, update more rapidly, and can handle more terms. It can also serve as a tool for KOS builders.

#### Strategic Space for Neuroscience Researchers

Our research provided a useful strategic space for neuroscience researchers. The results of clustering provide systematic lists of cognitive functions, imaging protocols, descriptive terms, brain structures, behavioral paradigms, diseases, algorithms, cell types, and genes. The relation between methods and keywords can help scientists find new research projects. The popular combinations suggest successful application while obscure combinations may reflect subjects that have potential for fruitful research. Research in neuroscience has evolved to become so complex that it is challenging to obtain an overall perspective of the field without using natural language processing algorithms and other tools. Our research here provided a preliminary view of the field.

### Limitations and Future Work

The results of word representation training and clustering were not thoroughly examined. All parameters were used from past research. However, if we want to obtain a high-quality category of terms, we need to optimize the settings and use well-known objective methods to examine its quality. Furthermore, the binary tree structure cannot adequately depict the hierarchical relationship between terms. We need to either modify the algorithm or invest more manual work to add links to the category of terms. Moreover, this term system cannot help users without being built into a web application. Systematic user evaluation will be conducted in future studies.

## Data Availability Statement

Publicly available datasets were analyzed in this study. This data can be found here: https://www.ncbi.nlm.nih.gov/pmc/; with accession numbers in Supplementary Data Sheet S7.

## Author Contributions

YZ was involved in setting the framework of this research. YW provided opinions on algorithm and data visualization methods. CH designed and implemented the algorithm. All authors were involved in writing and refining this manuscript.

## Conflict of Interest

The authors declare that the research was conducted in the absence of any commercial or financial relationships that could be construed as a potential conflict of interest.

The reviewer G-ZW declared a shared affiliation, though no other collaboration, with one of the authors, YZ, to the handling editor at time of review.

## Footnotes

1 https://meshb.nlm.nih.gov/treeview

2 https://meshb.nlm.nih.gov/record/ui?ui=D003071

3 https://www.ncbi.nlm.nih.gov/pmc/advanced
