# Supplementary material for: Creating Neuroscientific Knowledge Organization System Based on Word Representation and Agglomerative Clustering Algorithm
Source: Front Neuroinform. 2020 Aug 18;14:38. doi: 10.3389/fninf.2020.00038 (PMC7461893; doi:10.3389/fninf.2020.00038)
Supplement: Supplementary file 11 [file Presentation_1.pptx]

## Slide 1
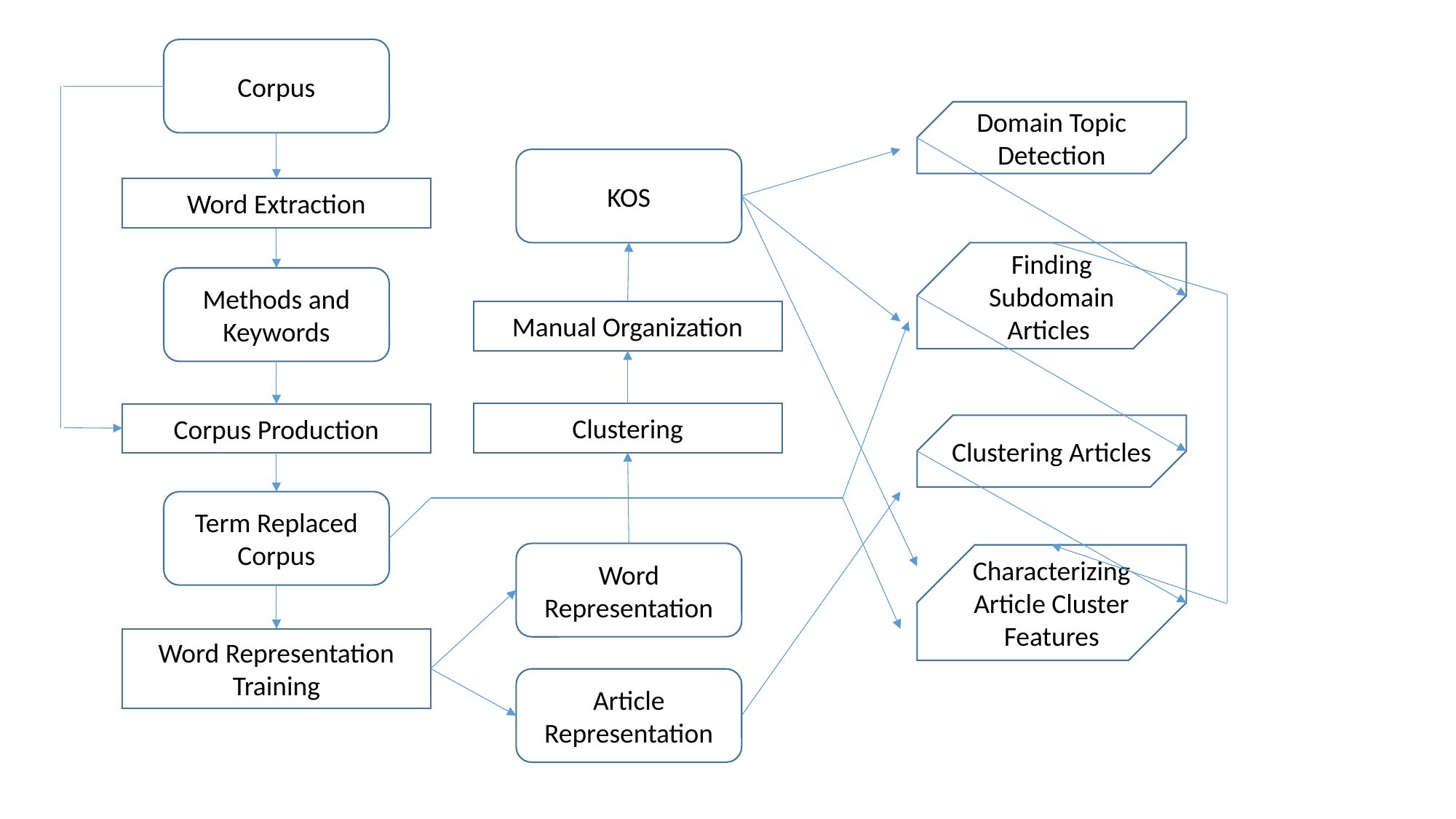

Corpus
Domain Topic Detection
KOS
Word Extraction
Finding Subdomain Articles
Methods and Keywords
Manual Organization
Clustering
Corpus Production
Clustering Articles
Term Replaced Corpus
Word Representation
Characterizing Article Cluster Features
Word Representation Training
Article Representation

## Slide 2
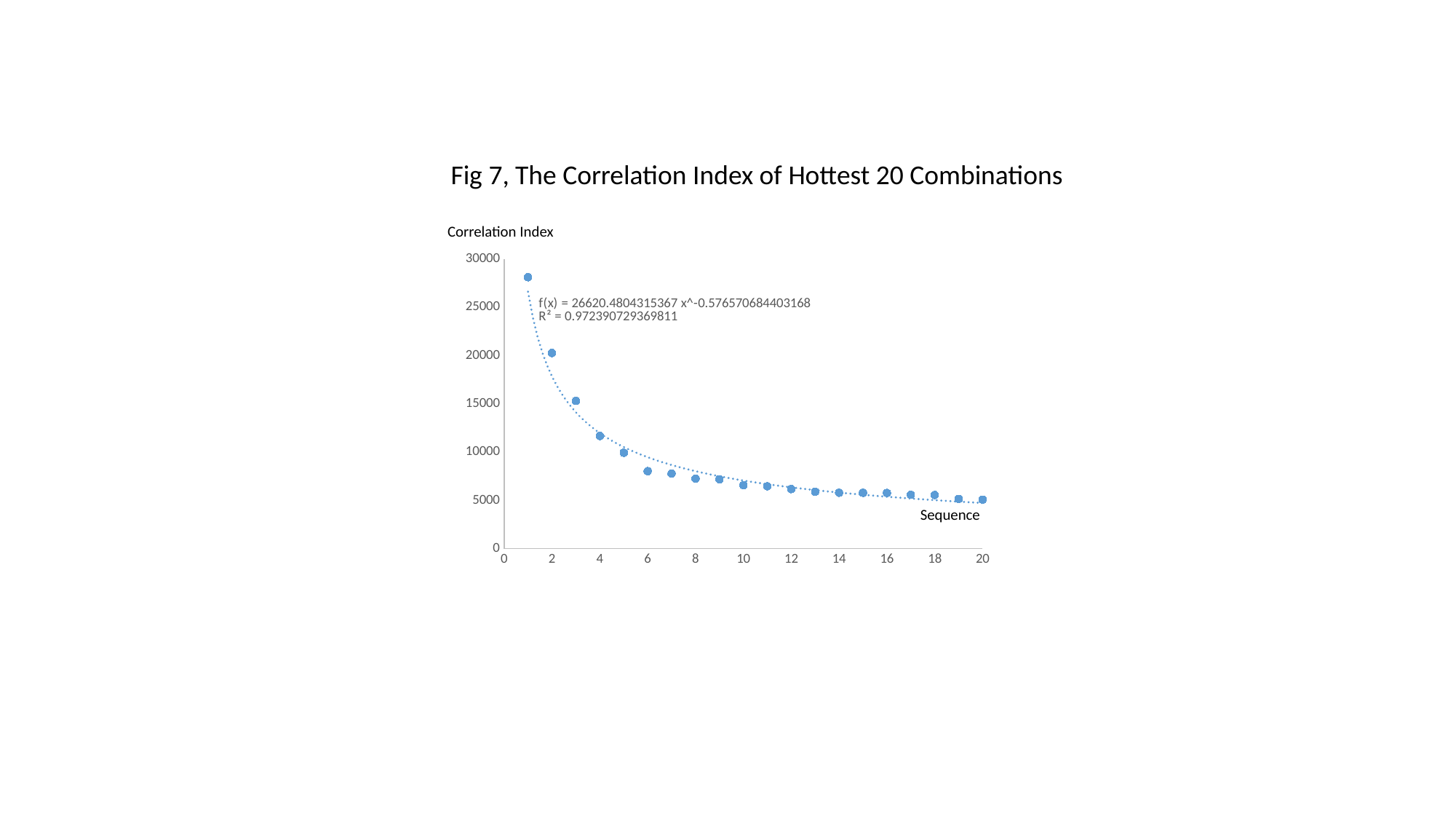

Fig 7, The Correlation Index of Hottest 20 Combinations
Correlation Index
### Chart
| Category | |
|---|---|Sequence

## Slide 3
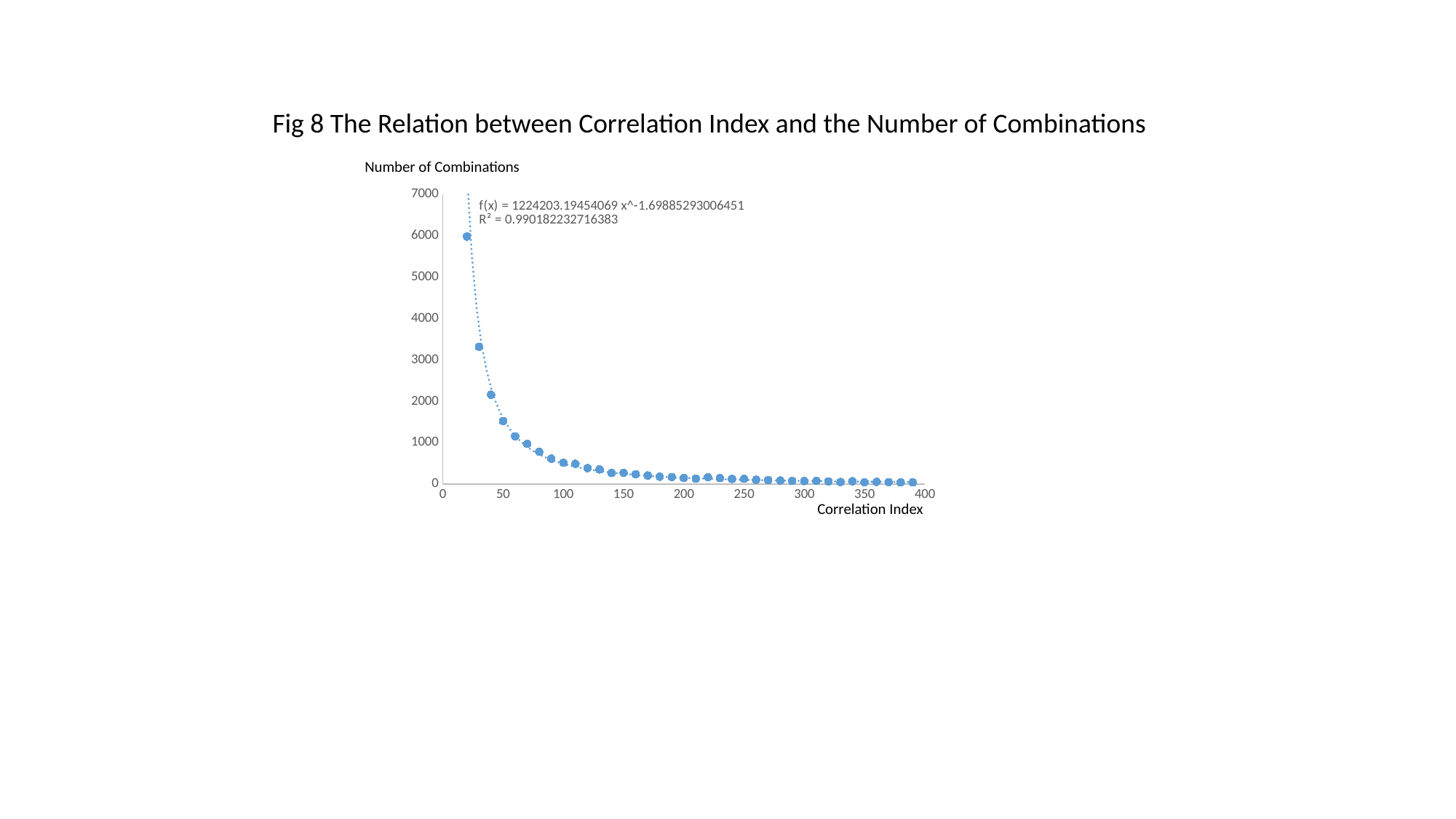

Fig 8 The Relation between Correlation Index and the Number of Combinations
Number of Combinations
### Chart
| Category | |
|---|---|Correlation Index

## Slide 4
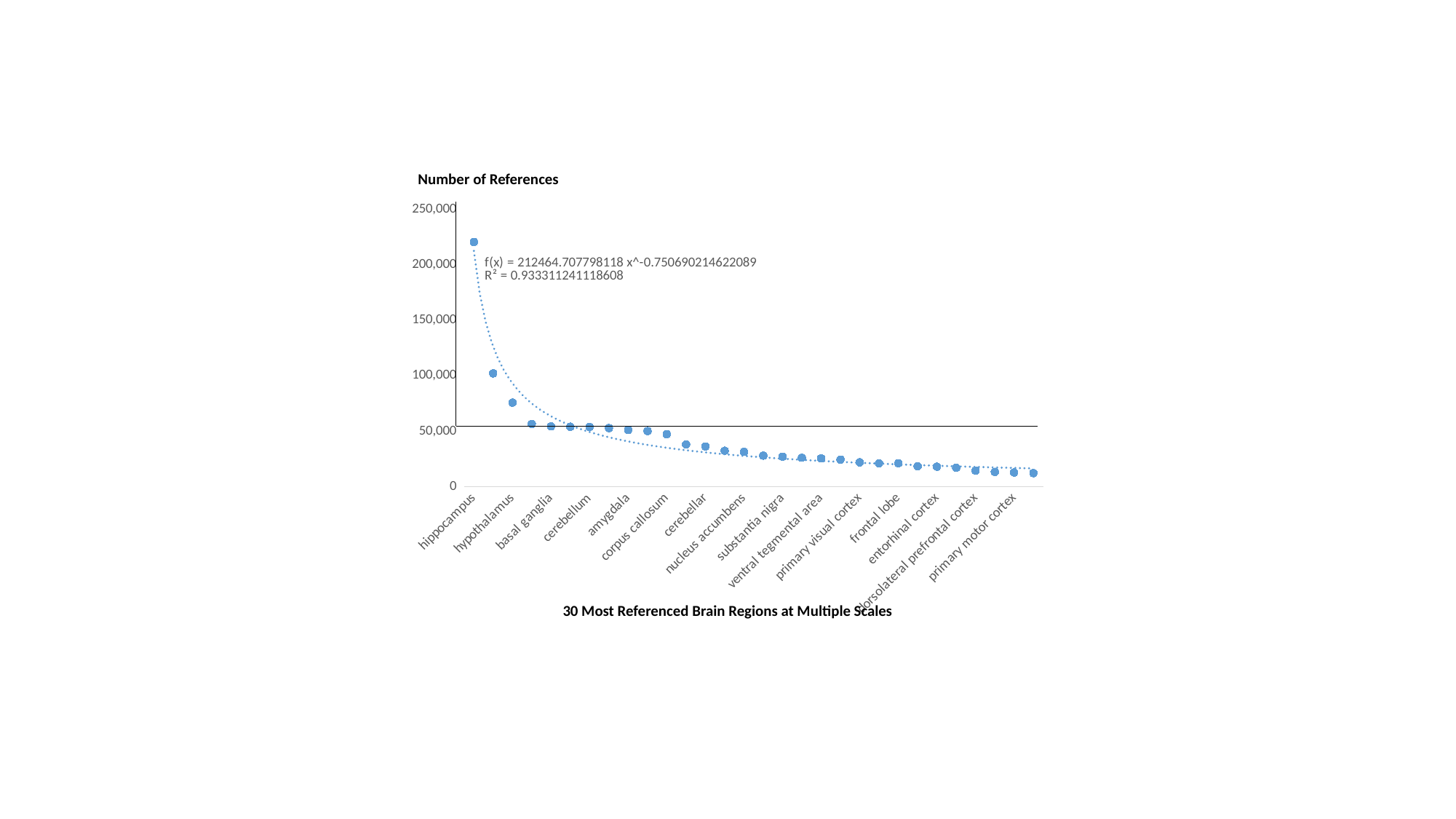

Number of References
### Chart
| Category | |
|---|---|
| hippocampus | 220503.0 |
| prefrontal cortex | 102151.0 |
| hypothalamus | 75833.0 |
| brain stem | 56607.0 |
| basal ganglia | 54403.0 |
| frontal cortex | 54064.0 |
| cerebellum | 53849.0 |
| anterior cingulate cortex | 52822.0 |
| amygdala | 51210.0 |
| visual cortex | 50199.0 |
| corpus callosum | 47333.0 |
| motor cortex | 38130.0 |
| cerebellar | 36285.0 |
| striatum | 32473.0 |
| nucleus accumbens | 31484.0 |
| thalamus | 28085.0 |
| substantia nigra | 27028.0 |
| temporal lobe | 26162.0 |
| ventral tegmental area | 25630.0 |
| medial prefrontal cortex | 24524.0 |
| primary visual cortex | 22008.0 |
| ca1 | 21194.0 |
| frontal lobe | 21184.0 |
| medial temporal lobe | 18427.0 |
| entorhinal cortex | 18087.0 |
| parietal cortex | 17077.0 |
| dorsolateral prefrontal cortex | 14521.0 |
| orbitofrontal cortex | 13397.0 |
| primary motor cortex | 12870.0 |
| medial prefrontal cortex (mpfc) | 12298.0 |30 Most Referenced Brain Regions at Multiple Scales
