# Supplementary figures and images for: Creating Neuroscientific Knowledge Organization System Based on Word Representation and Agglomerative Clustering Algorithm
Source: Front Neuroinform. 2020 Aug 18;14:38. doi: 10.3389/fninf.2020.00038 (PMC7461893; doi:10.3389/fninf.2020.00038)

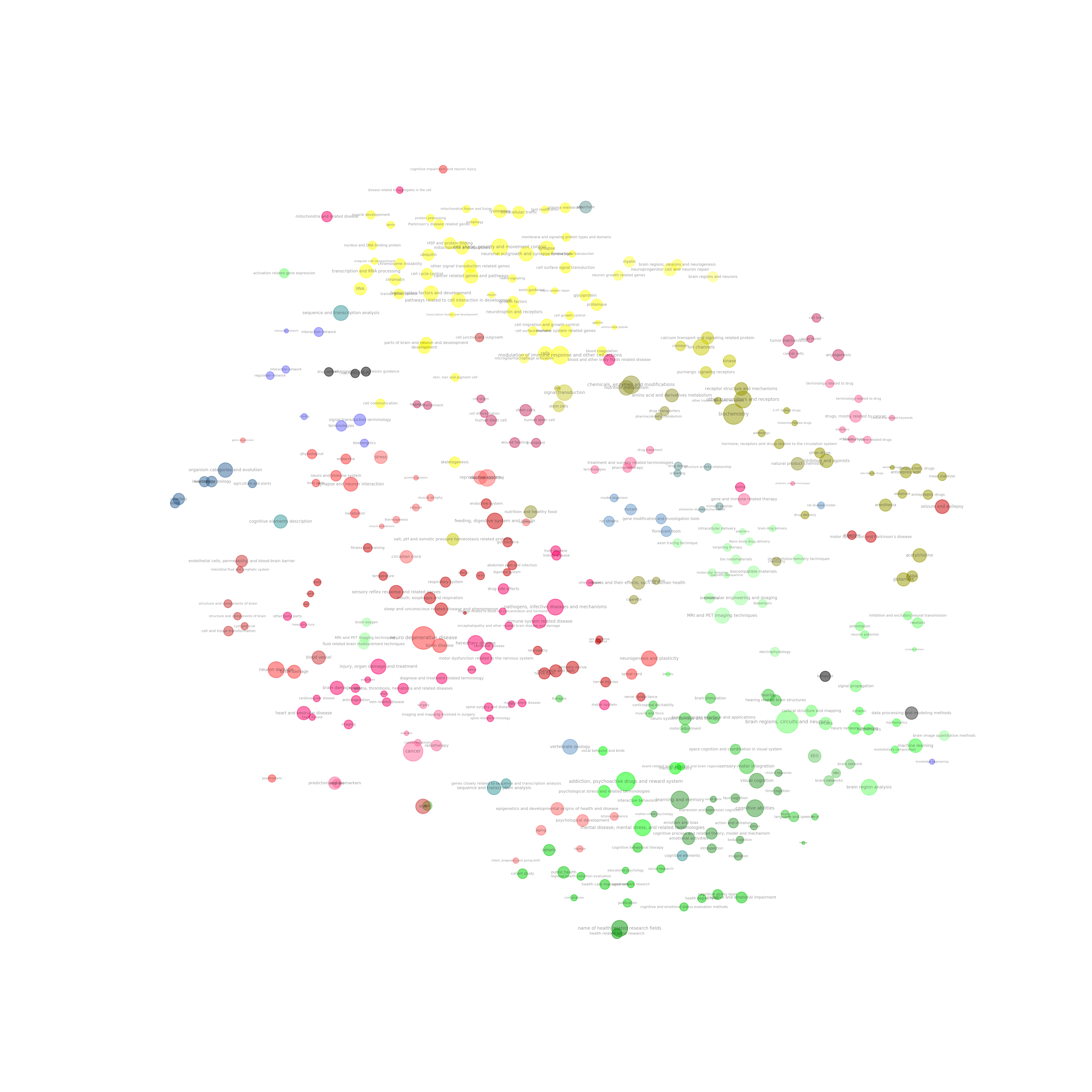

Supplement: Supplementary file 12 [file Image_1.JPEG]

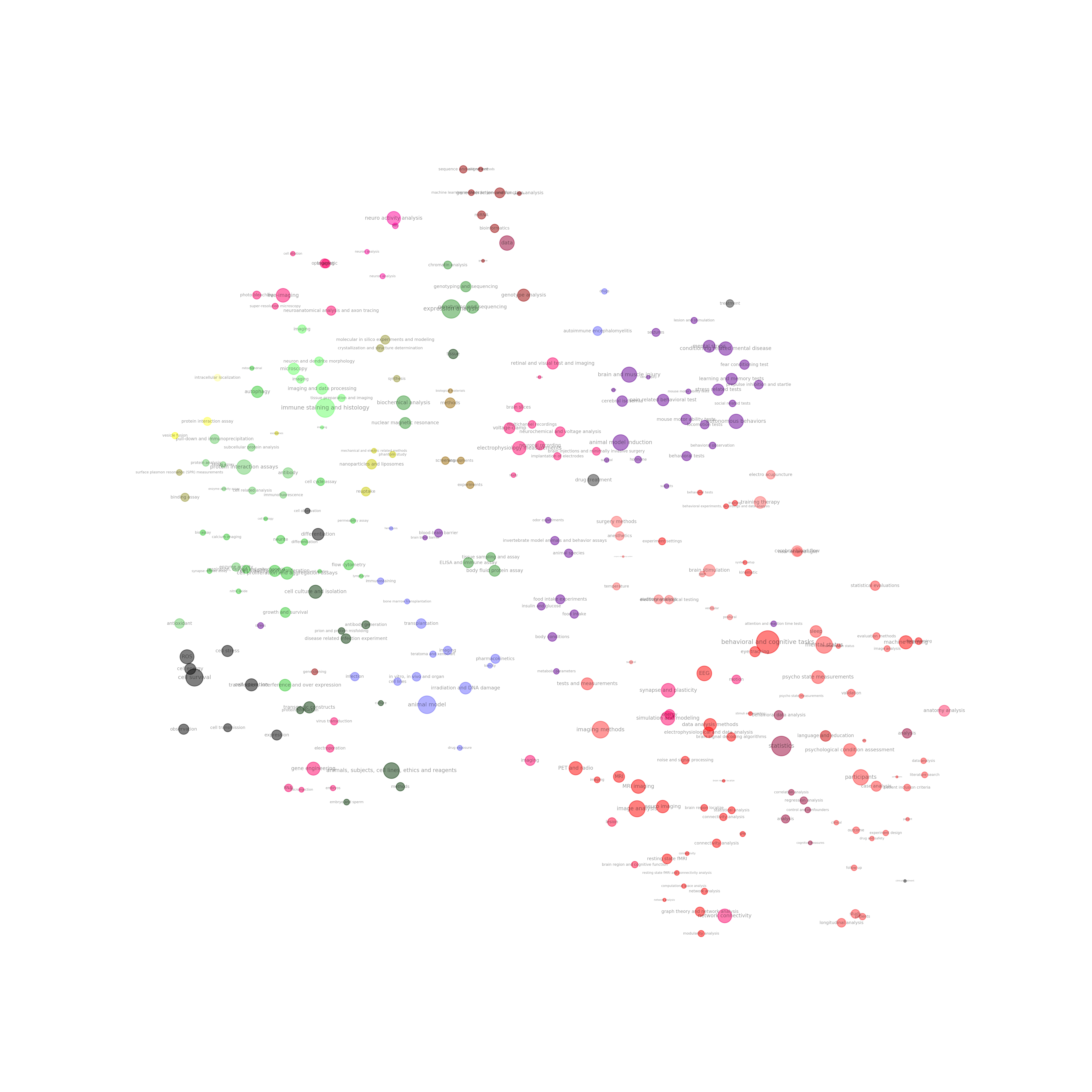

Supplement: Supplementary file 13 [file Image_2.JPEG]
